# Simple, fast and inexpensive quantification of glycolate in the urine of patients with primary hyperoxaluria type 1

**DOI:** 10.1007/s00240-023-01426-6

**Published:** 2023-03-15

**Authors:** Thomas Boehm, Cristina Martin-Higueras, Eva Friesser, Clara Zitta, Silvia Wallner, Adam Walli, Katarina Kovacevic, Holger Hubmann, Kristaps Klavins, Peter Macheroux, Bernd Hoppe, Bernd Jilma

**Affiliations:** 1 Department of Clinical Pharmacology, Medical University of Vienna, Waehringer Guertel 18-20, 1090 Vienna, Austria; 2 German Hyperoxaluria Center, Bonn, Germany; 3 Institute of Biochemistry, Graz University of Technology, Graz, Austria; 4 Laboratory Dr. Wisplinghoff, Forensic and Clinical Toxicology, Cologne, Germany; 5 Department of Paediatrics and Adolescent Medicine, Division of General Paediatrics, Medical University of Graz, Graz, Austria; 6 Rudolfs Cimdins Riga Biomaterials Innovations and Development Centre, Institute of General Chemical Engineering, Faculty of Materials Science and Applied Chemistry, Riga Technical University, Riga, Latvia

**Keywords:** Primary hyperoxaluria type 1, Glycolate, Glyoxylate, Recombinant glycolate oxidase, Ortho-aminobenzaldehyde

## Abstract

In primary hyperoxaluria type 1 excessive endogenous production of oxalate and glycolate leads to increased urinary excretion of these metabolites. Although genetic testing is the most definitive and preferred diagnostic method, quantification of these metabolites is important for the diagnosis and evaluation of potential therapeutic interventions. Current metabolite quantification methods use laborious, technically highly complex and expensive liquid, gas or ion chromatography tandem mass spectrometry, which are available only in selected laboratories worldwide. Incubation of ortho-aminobenzaldehyde (oABA) with glyoxylate generated from glycolate using recombinant mouse glycolate oxidase (GO) and glycine leads to the formation of a stable dihydroquinazoline double aromatic ring chromophore with specific peak absorption at 440 nm. The urinary limit of detection and estimated limit of quantification derived from eight standard curves were 14.3 and 28.7 µmol glycolate per mmol creatinine, respectively. High concentrations of oxalate, lactate and L-glycerate do not interfere in this assay format. The correlation coefficient between the absorption and an ion chromatography tandem mass spectrometry method is 93% with a *p* value < 0.00001. The Bland–Altmann plot indicates acceptable agreement between the two methods. The glycolate quantification method using conversion of glycolate via recombinant mouse GO and fusion of oABA and glycine with glyoxylate is fast, simple, robust and inexpensive. Furthermore this method might be readily implemented into routine clinical diagnostic laboratories for glycolate measurements in primary hyperoxaluria type 1.

**Supplementary Information:**

The online version contains supplementary material available at 10.1007/s00240-023-01426-6.

## Introduction

The primary hyperoxalurias (PH) are rare inherited metabolic diseases of the glyoxylate metabolism in the liver which display variable age of clinical onset (median age at onset of 5.5 years) [1]. Primary hyperoxaluria type 1 (PH1; OMIM 259,900) is caused by mutations in the liver-specific peroxisomal enzyme alanine-glyoxylate aminotransferase and is responsible for 80% of PH cases and accounts for 1–2% of pediatric end-stage renal disease patients [1–3]. At the time of first diagnosis 20–50% of patients already suffer from advanced kidney disease. The PH1 enzyme deficiency results in the accumulation of glyoxylate and consequently excessive production of oxalate and glycolate. Glyoxylate appeared to be unstable in urine and cannot be readily used for quantification and diagnosis [4]. Urinary oxalate excretion is increased in all three PH variants with variable urinary concentrations unsuitable for diagnostic purposes. Since glycolate concentrations are not elevated in all suspected PH1 cases the definite diagnosis is derived from genetic testing in addition to glycolate measurements [1, 5].

State-of-the-art quantification methods of glycolate in urine and tissue samples are based on ion, gas or liquid chromatography tandem mass spectrometry (IC-, GC-, LC–MS/MS) approaches partially involving labor-intensive urine extraction steps using organic solvents [6–11]. Quantification of glycolate is not readily available in routine clinical laboratories, both because of the expensive equipment needed and the expertise required to operate these highly technological instruments [12]. Only a few laboratories worldwide routinely quantify glycolate to support the diagnosis of primary hyperoxaluria. Thus, an easier method available in more clinical laboratories might help in the diagnosis, and the evaluation of treatment modalities.

Fusion of *ortho*-aminobenzaldehyde (oABA) with aldehydes like glyoxylate generates a chromophore with a proposed dihydroquinazoline double aromatic ring structure [13–15]. Glyoxylate can be generated using glycolate oxidase (GO). The oxidation of glycolate using GO releases hydrogen peroxide and GO was already used for the quantification of glycolate in urine samples [16–19]. Nevertheless, urine like plasma contains many hydrogen peroxide trapping anti-oxidative substances negatively influencing any assay type that relies on the quantification of hydrogen peroxide [20]. Pre-assay steps like charcoal adsorption are necessary to partially remove the interfering substances [16]. Petrarulo et al. [4, 21] circumvented the hydrogen peroxide-trapping problem by pre-column derivatization of GO-generated glyoxylate with phenylhydrazine and co-incubation with cysteine. Reverse phase high pressure liquid chromatography (rpHPLC) was necessary for detection. Another disadvantage is that phenylhydrazine seems to inhibit GO activity which requires the use of very high GO concentrations.

Here we show that the absorption properties of the condensation product between GO-generated glyoxylate, high concentrations of glycine and oABA allow simple, fast, robust and inexpensive quantification of glycolate concentrations in the urine of healthy individuals and PH1 patients. Urine does not need to be pre-treated and no chromatography steps are required. The only necessary equipment is a spectrophotometer or a microplate reader with a sensitive absorption measurement module.

## Materials and methods

### Generation of the dihydroquinazoline chromophore CCMDQ for mass spectrometry analysis

**Fig. 1:**
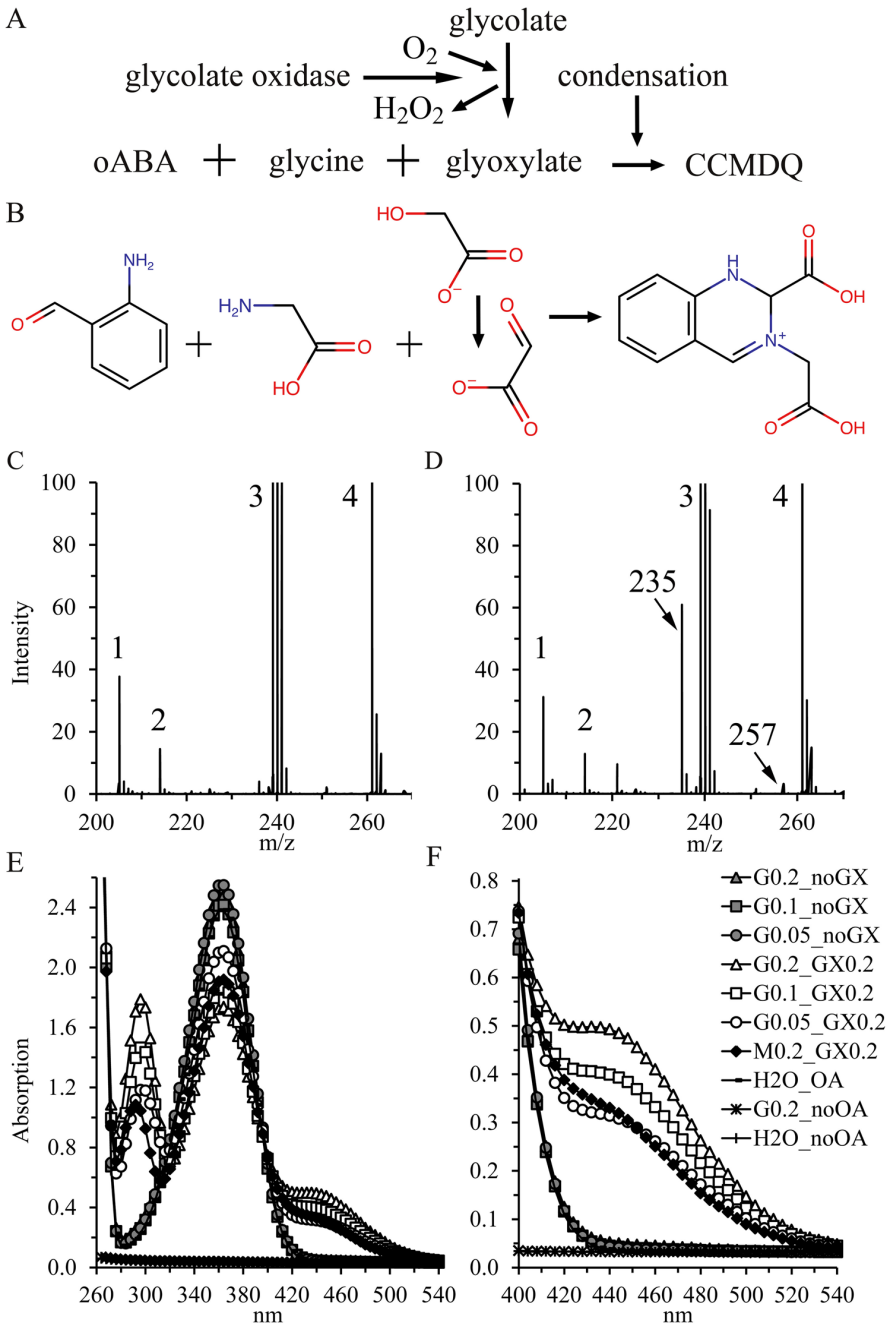
Generation and characterization of the dihydroquinazoline chromophore CCMDQ. **A** After oxidation of glycolate (2-hydroxyacetate) using glycolate oxidase the reaction product glyoxylate (oxaldehydrate) condenses in the presence of high glycine concentrations (200 mM) and *ortho*-aminobenzaldehyde (oABA) generating the chromophore 2-carboxy-3-(carboxymethyl)-1,2-dihydroquinazolin-3-ium (CCMDQ); The release of H_2_O and OH^−^ during condensation are not shown; **B** Chemical structures; **C**, **D** Mass spectrometry data after incubation of oABA and glycine in the absence (C) or presence (D) of glyoxylate (GX); The major peaks are at 205 m/z (1); 214 m/z (2) and derived from hepes (3 = 239, 240, 241 m/z) and its sodium adducts (4 = 261, 262, 263); Glycine and oABA are detected but not shown; In the presence of glyoxylate a strong peak at 235 Da appeared representing CCMDQ; The likely sodium adduct at 257 Da (+ 22 Da) was also detected; A signal at 236 m/z is present in both samples; Fragmentation analysis is shown in Supplementary Fig. S1 and Table S1; **E**,** F** Absorption scans after incubation of oABA (1 mM), different glycine concentrations (200, 100 and 50 mM) with GX (200 µM) or methylamine (200 µM) and adequate controls without glycine (G), oABA (OA) and GX; **F** represents an enlargement of the relevant absorption area between 400 and 540 nm; *M* methylamine

The International Union of Pure and Applied Chemistry (IUPAC) name for the proposed condensate of oABA with glycine and glyoxylate is 2-carboxy-3-(carboxymethyl)-1,2-dihydroquinazolin-3-ium with the abbreviation CCMDQ (Fig. 1A, B). For electrospray ionization MS analysis (ESI–MS) a final concentration of 0 or 300 µM glyoxylate (G4502; freshly prepared as 6 mM stock solution in water) was incubated in 5 mM hepes buffer (H3375) pH 7.0 with a final concentration of 1 mM oABA (A9628) and 100 mM ultrapure glycine (94,119) for 30 min at room temperature. Ultrapure glycine showed the lowest background and highest signal over background ratio (data not shown). If not otherwise indicated chemicals were purchased from Sigma-Aldrich (St. Louis, MO). oABA was dissolved in 100% ethanol and stored for approximately 6 months at − 32 °C without an increase in polymerization or a noticeable reduction of performance (data not shown). The two samples with and without glyoxylate were frozen at − 32 °C for less than 2 weeks. An aliquot was used for absorption scan analysis and extinction coefficient calculations.

The dihydroquinazoline structure of the condensate between oABA, glycine and glyoxylate was predicted but not analyzed using nuclear magnetic resonance (NMR) or MS. Samples with and without glyoxylate were analyzed using direct infusion MS. A Xevo TQ-MS mass spectrometer equipped with ESI was employed for the analysis. More details about the ESI–MS method to detect CCMDQ are provided in the Supplement.

### Absorption scan of CCMDQ

Different concentrations of ultrapure glycine (200, 100 and 50 mM) or 200 mM methylamine (M0505), and 1 mM oABA were incubated in 50 mM hepes buffer for 30 min in the dark with or without 200 µM glyoxylate. Absorption was measured in a Synergy^™^ H1 multi-mode microplate reader (BioTek, Winooski, VT) and scans were performed using 4 nm steps. The resolution of the Synergy^™^ H1 microplate reader for absorbance measurements is 0.0001 optical density units.

### Generation of CCMDQ using glyoxylate, glycine and oABA in three urine samples from healthy individuals

After thawing all urine samples were centrifuged at high speed (16,000*g*) for 5 min at 4 °C to remove possible cell debris and aggregates. The supernatants of the urine samples from three healthy volunteers (HV) were diluted with water reaching a final creatinine concentration of 4.0 mM. Hepes buffer (50 mM, pH 7.0) is able to adequately buffer most urine samples. The mean pH (± standard deviation, SD) of seven urine samples with 50 mM hepes was 6.85 (0.25). Final glyoxylate concentrations were 0, 3.7, 11, 33, 100 and 300 µM. The final oABA and ultrapure glycine concentrations were 2 mM and 100 mM, respectively. The samples were incubated at room temperature in the dark for 60 min and absorption at 440 nm was measured every 10 min.

### Expression and purification of recombinant mouse glycolate oxidase (GO)

Mouse glycolate oxidase (mGO, Q9WU19, Hao1, EC 1.1.3.15) was expressed and purified in *E. coli* using standard procedures [22]. Expression and purification details are described in the Supplement.

### Measurement of glycolate concentrations in urine using GO, glycine and oABA

Glycolate concentrations in all standard curves, urine samples from healthy individuals and the different PH samples were measured using urine adjusted to 1 mM creatinine. Glycolate standard was purchased from Sigma-Aldrich (CDS000626). The final assay format was composed of 50 mM hepes pH 7.4, 200 mM ultrapure glycine and 4 mM oABA with and without 1 µM GO in duplicate. Urinary glycine concentrations are between 0.1 and 4 mM and therefore, at least 50-fold lower compared to the added ultrapure glycine [23, 24]. Endogenous glycine will not relevantly interfere in the assay format. The condensation reaction is stable between pH 5 and pH 8.8 to within 90% compared to pH 7.4 [15].

All urine samples were incubated in the dark in duplicate at 37 °C for 90 min with absorption measurements at 440 nm every 2.5 min. The mean absorption at 440 nm of the duplicates without GO was subtracted from the mean with GO to obtain the GO-specific signal. The GO auto-absorption of 0.0120 was subtracted from this specific signal and the area under the curve (AUC) comprising 37 time points was calculated using the “sumproduct” formula in Microsoft Excel. Four parameter logistic (PL) non-linear regression analyses were used to calculate the standard curves and corresponding glycolate concentrations.

### Testing the interference of oxalate, glycerate and lactate during GO-mediated glycolate to glyoxylate conversion

The same assay format as described above was used with 250 and 500 µM sodium glycolate (CDS000626), sodium oxalate (379,735), sodium lactate (L7022) and sodium L-glycerate (51,738). In the second set of experiments 250 µM sodium glycolate was simultaneously incubated with different concentrations of sodium oxalate, lactate and L-glycerate (250, 125, 63 µM) to test direct inhibition or substrate competition with glycolate. The selected oxalate (> 12 years; 2–80 µmol/mmol creatinine), lactate (> 18 years; 3.6–11 µmol/mmol creatinine) and L-glycerate (> 5 years; 22–123 µmol/mmol creatinine) concentrations are at least 2- to tenfold above normal levels [1, 25].

### Testing of glycolate concentrations in healthy children, adults and PH patients

The same assay format as described above was also used for urine samples from HV and PH patients. These four cohorts are briefly described in the Supplement (Supplementary Tables S4–S7). In the main manuscript, glycolate concentrations from Cohort_0_HV and Cohort_3_PH are presented. Thirteen (81%) of the 16 PH1 patients in Cohort_3_PH were treated with 8–11 mg/kg/day pyridoxine. The spot unpreserved urine samples were immediately frozen after collection and measured within 6 months using the same assay protocol, reagents and control urine for establishing the glycolate standard curve.

### Measurement of glycolate using mass spectrometry

Glycolate measurements for Cohort_3_PH were performed via IC-MS on a Dionex Integrion HPIC coupled with an ISQ EC single quadrupole mass spectrometer. Briefly, urine collected at home over 24 h was acidified with 6 mM HCl and transported to the laboratory within a few days. After arrival samples were analyzed within 24 h in most cases. Glycolate was detected using selected ion monitoring (SIM) at 75 m/z and quantified using a glycolate standard curve. Urine samples were diluted 1–100 in 0.3 M boric acid (B6768 BioReagent, ≥ 99.5%, Sigma-Aldrich). The glycolic acid standard curve consisted of glycolic acid at 0, 0.2, 0.4, 1, 2 and 4 µM. Two quality control samples at 1.2 and 3 µM glycolate in 0.3 M boric acid were used. Details about the measurement method are described in the Supplement.

### Ethics

All procedures were in accordance with the ethical standards of the responsible committee on human experimentation (institutional and national) and with the Helsinki Declaration of 1975, as revised in 2013. The ethics committees in Graz permitted the use of the biological material without a formal vote because the urine samples of healthy children and adolescents were completely and irreversibly anonymized and collected during routine out-patient visits for medical reasons unrelated to PH. Samples from hyperoxaluria patients were collected under the ethics committee numbers Bonn 113/14 und Aekno 2021473. The study numbers for the collection of some urine samples from healthy adult volunteers in Vienna are EK:2030/2013 and EK:1810/2015. All patients (or their parents) and all HV (or their parents) provided their informed consent before the collection of urine samples.

### Statistics

Calculations of the 95% prediction intervals in Fig. 3A were performed using the means of *n* = 57 control samples with *n*−1 degrees of freedom (*f*) and the quantile *t* distribution to account for the estimation of the SD. The 95% prediction intervals were derived using the mean + 2.0 (*n* = 57) * SD of the mean * √(1 + 1/*n*).

The signals of the glycolate standards were used to calculate the glycolate concentrations via 4PL non-linear regression using OriginPro 8.5 software (OriginLab Corporation, Northampton, MA). The limit of blank (LoB) cannot be calculated because of endogenous glycolate. The limit of detection (LoD) was calculated using the following formula: 3.55 [= c_beta calculated using 3.49/(1–1/4*f); 99% *t*-distribution] × weighted SDs of the 16 and 31 µM glycolate standards [26]. We always used urine at 1 mM creatinine and therefore 1 µM glycolate corresponds to 1 µmol glycolate per mmol creatinine. If in a few cases the urine creatinine concentration was below 1 mmol/L, urine was used undiluted and the calculated glycolate concentration extrapolated to 1 mmol/L creatinine. The estimated limit of quantification (eLoQ) was calculated using the LoD + 3.55 [= c_beta; 99% *t*-distribution] * weighted SDs of the 16 and 31 µM glycolate standard.

Testing for normal distribution was performed using both the more conservative Shapiro–Wilk and the Kolmogorov–Smirnov test. Outliers were determined using the Grubbs outlier test. The Bland–Altmann plot was used to measure the limit of agreements (LOA) between the mouse recombinant GO/glycolate conversion assay method and the MS method [27, 28].

## Results

### Generation and absorption properties of the dihydroquinazoline fusion product CCMDQ

Fusion of aldehydes including glyoxylate, glycine and oABA in buffer matrices has been previously described, but the proposed chemical structure and corresponding molecular weight was never verified using MS (Fig. 1) [13–15]. Incubation of glyoxylate with glycine and oABA generated a m/z signal at the predicted mass of 235 Da only in the presence of glyoxylate (Fig. 1C, D). The likely sodium adduct at 257 Da was also detected. Fragmentation MS2 analysis of the 235 m/z signal is presented in the Supplement (Supplementary Fig. S1; Supplementary Table S1). Several predicted fragments were identified strongly indicating the correctness of the proposed dihydroquinazoline structure. The absorption scan from 260 to 540 nm showed dose-dependent glyoxylate-specific peak signals at 295 and 440 nm (Fig. 1E, F). The signal at 440 nm (Fig. 1F) is preferred for quantification, because of the lower background at > 400 nm compared to 295 nm in complex matrices. The extinction coefficient of CCMDQ was 2219 M^−1^ cm^−1^ (Supplementary Fig. S1A). We also tested methylamine instead of glycine, but the peak signal was consistently lower (Fig. 1E, F; data not shown). Condensation of oABA with delta-1-pyrroline (THPQ) or delta-1-piperideine (HHPQ), the autocyclization products of putrescine or cadaverine after deamination using diamine oxidase, generates triple aromatic ring structures with absorption maxima at 430 and 460 nm respectively. Both chromophores are also fluorophores with strong fluorescence at 620 nm after excitation at the absorption maxima [20]. No relevant fluorescence emission could be detected using emission and excitation scan analysis of CCMDQ (Supplementary Figs. S2 and S3). The extinction coefficients or absorption maxima of HHPQ (2242 M^−1^ cm^−1^), THPQ (1860 M^−1^ cm^−1^) and CCMDQ (2219 M^−1^ cm^−1^) are similar implying that the third aromatic ring is necessary for fluorescence.

### Urine is amenable for the measurement of the oABA/glyoxylate/glycine condensation product CCMDQ

**Fig. 2:**
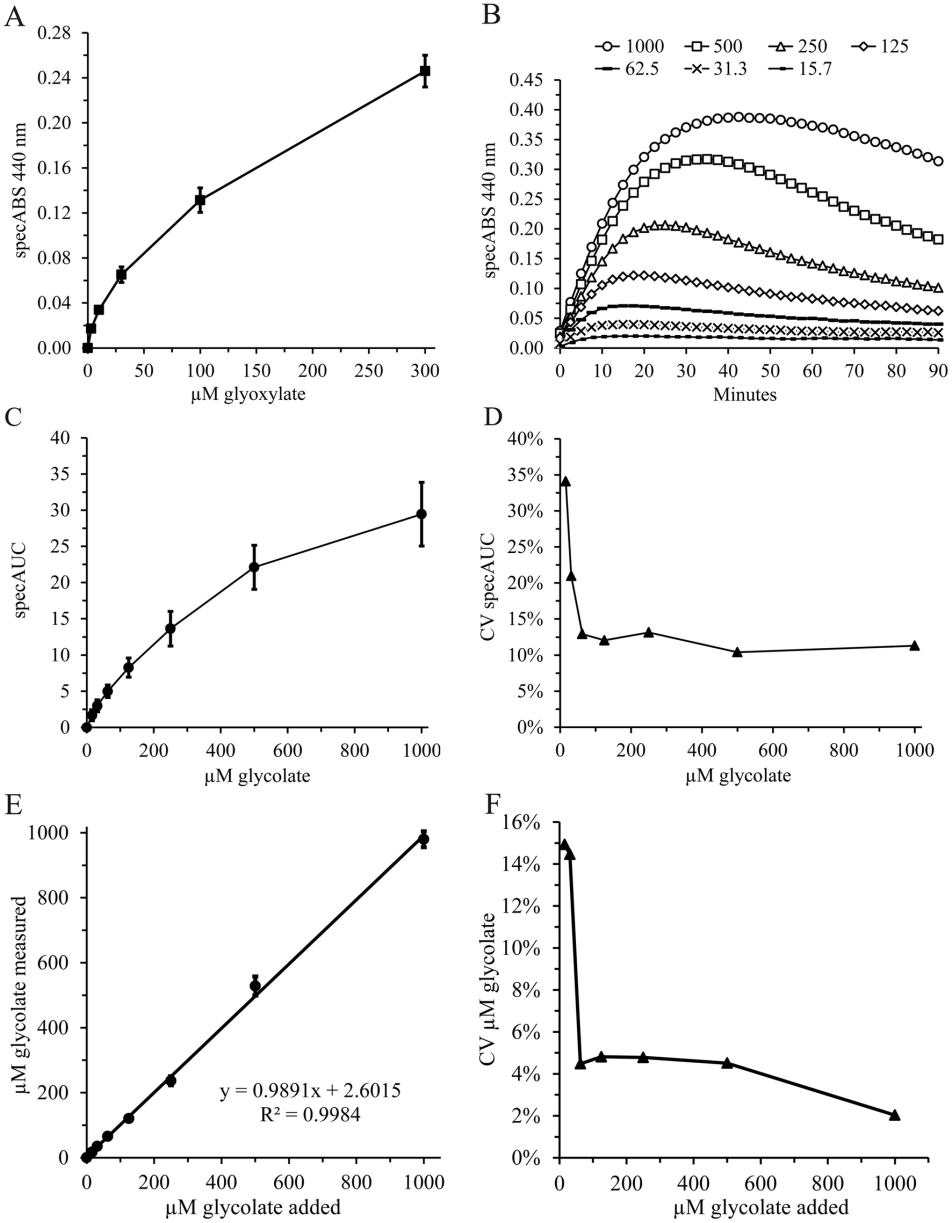
In human urine recombinant mouse glycolate oxidase efficiently converts glycolate into glyoxylate with rapid generation of CCMDQ. **A** Different glyoxylate concentrations were spiked into urine samples of three healthy volunteers (HVs) in the presence of 4 mM oABA and the peak absorption at 440 nm was normalized to the absorption without the addition of exogenous glyoxylate; Samples were measured in duplicate; The curve represents the specific mean of the three curves including the upper and lower 95% confidence intervals (CI; *n* = 3; *t*-value 4.303); The time course of the absorption signal is shown in Supplementary Fig. S4; **B** Different glycolate concentrations (15.7, 31.3, 62.5, 125, 250, 500 and 1000 µM) with 4 mM oABA and 1 µM recombinant mouse glycolate oxidase (GO) were incubated for 90 min at 37 °C and absorption at 440 nm was measured every 2.5 min; The curves represent the mean specific absorption at 440 nm during the 90 min incubation period of eight independent experiments using urine from a HV; Samples were measured in duplicate; **C** The curve represents the mean specific AUC of the different added glycolate concentrations including the 99% CI of the eight standard curves shown in Panel B; **D** The CV of the mean and standard deviation of eight standard curves (Panel C) are shown dependent on the added glycolate (GLYC) concentrations; **E** The spiked glycolate concentrations are plotted against the mean of the measured glycolate concentrations calculated using the 4PL non-linear regression analysis from each of the eight standard curves; The error bars represent the 99% CI; **F** The CVs of Panel E are shown

Condensation of glycine, oABA and glyoxylate or other aldehydes were tested in buffer but not in complex matrices such as plasma or urine, which might contain potentially interfering substances. In the first experiment we spiked different concentrations of glyoxylate into urine samples of HV and measured specific peak absorption at 440 nm (Fig. 2A). The time course of the specific 440 nm absorption signal during the first 60 min is shown in Supplementary Fig. S4. After demonstrating that in urine glyoxylate can be readily fused with glycine and oABA generating CCMDQ, urine samples were spiked with recombinant mouse GO, glycine, oABA and different glycolate concentrations. The mean curve of eight standard curve determinations testing exogenous glycolate concentrations between 16 and 1000 µM are presented in Fig. 2B. The AUC method was used to calculate standard curves for quantification of glycolate concentrations in urine samples (Fig. 2C). The coefficients of variation (CVs) of the eight standard curves were below 15% using > 60 µM glycolate (Fig. 2D). The correlation coefficient between added and measured glycolate concentrations is > 99% with low CVs (Fig. 2E, F).

Using data from eight standard curves the LoD was 14.3 µmol glycolate per mmol creatinine and the eLoQ 28.7 µmol glycolate per mmol creatinine. The dynamic range of the assay is approximately 1 mmol glycolate per mmol creatinine using the LoD of 14.3 µmol glycolate per mmol creatinine as the lower and the highest standard of 1 mmol glycolate per mmol creatinine as the upper limit. Nevertheless, the standard curve between 500 and 1000 µM is flatter compared to the lower glycolate concentrations with likely increased imprecision. Higher sample dilutions in rare circumstances circumvent this issue.

### Lactate, oxalate and L-glycerate do not relevantly interfere in the GO assay

**Fig. 3:**
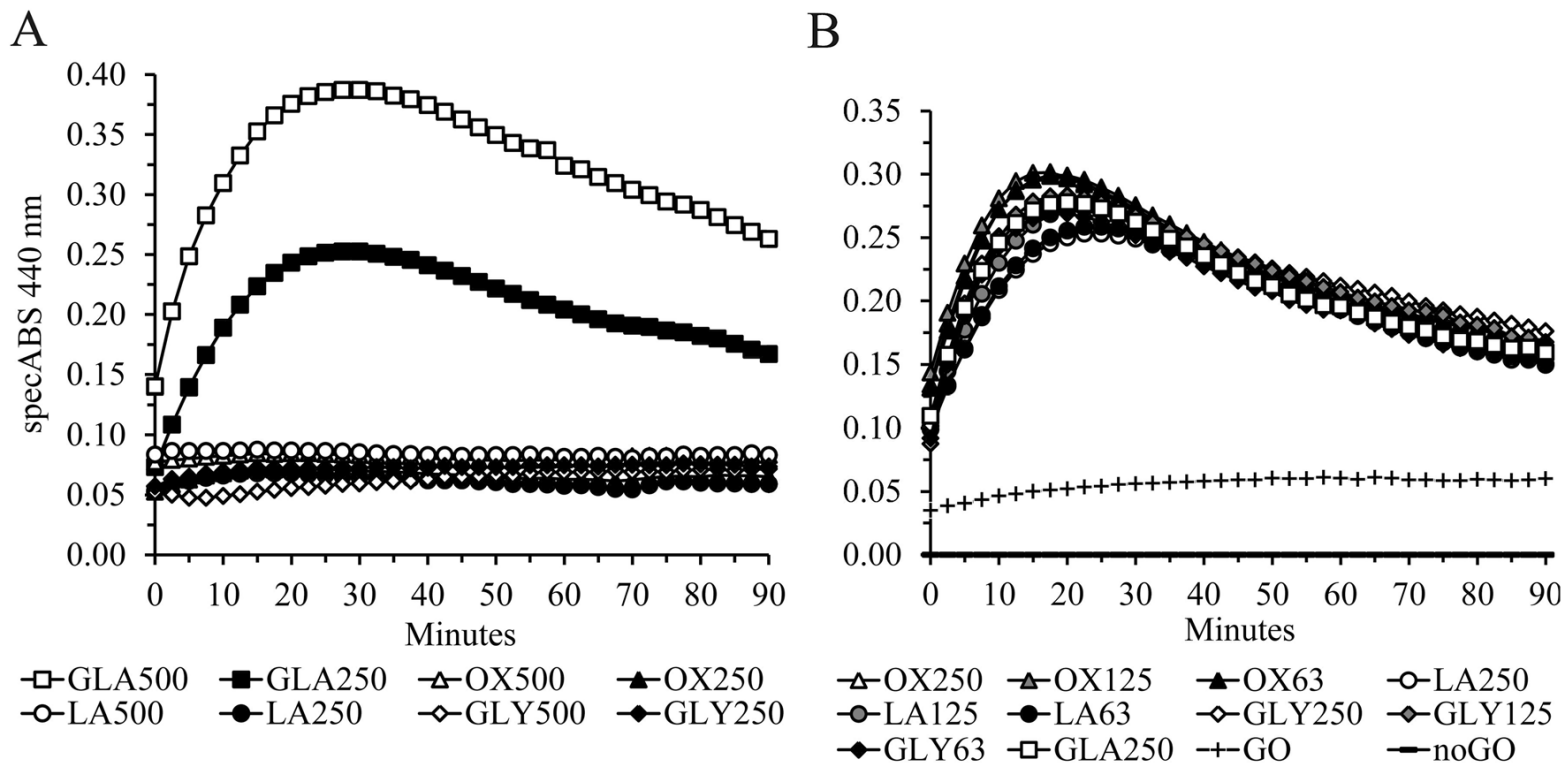
Oxalate, L-glycerate and lactate do not interfere in the glycolate oxidase assay. **A** Only glycolate (GLA) but not sodium oxalate (OX), sodium lactate (LA) or sodium L-glycerate (GLY) show specific absorption at 440 nm using 1 µM recombinant mouse glycolate oxidase (GO), 200 mM ultrapure glycine and 4 mM oABA in a urine sample from a healthy volunteer (HV); 250 and 500 represent final µM concentrations; **B** 63, 125 or 250 µM LA, OX or GLY do not influence the AUC absorption patterns using 250 µM GLA; The AUC deviations are shown in Supplementary Table S2; Data were normalized to the noGO curve containing no GO; The endogenous GLA concentration is represented by the ( +) GO curve; In both Panels A and B, the mean of duplicate measurements is shown; specABS = specific absorption

Oxalate concentrations can be highly elevated in the urine of PH patients and might interfere with glycolate/glyoxylate conversion. L-glycerate also accumulates in the urine of PH2 patients. Glycolate oxidase might also use lactate as a substrate and therefore we also tested lactate for interference [29]. High concentrations of L-glycerate, oxalate and lactate (250 and 500 µM) did not generate any specific absorption signal at 440 nm (Fig. 3A). Competition of three concentrations of oxalate, L-glycerate and lactate (63, 125 and 250 µM) do not significantly influence the AUC values using 250 µM glycolate (Fig. 3B). The mean (SD; *n* = 9) percent deviation was − 1.5% (5.1%; Supplementary Table S2).

### Measurement of glycolate concentrations in urine samples of healthy individuals and hyperoxaluria patients

**Fig. 4:**
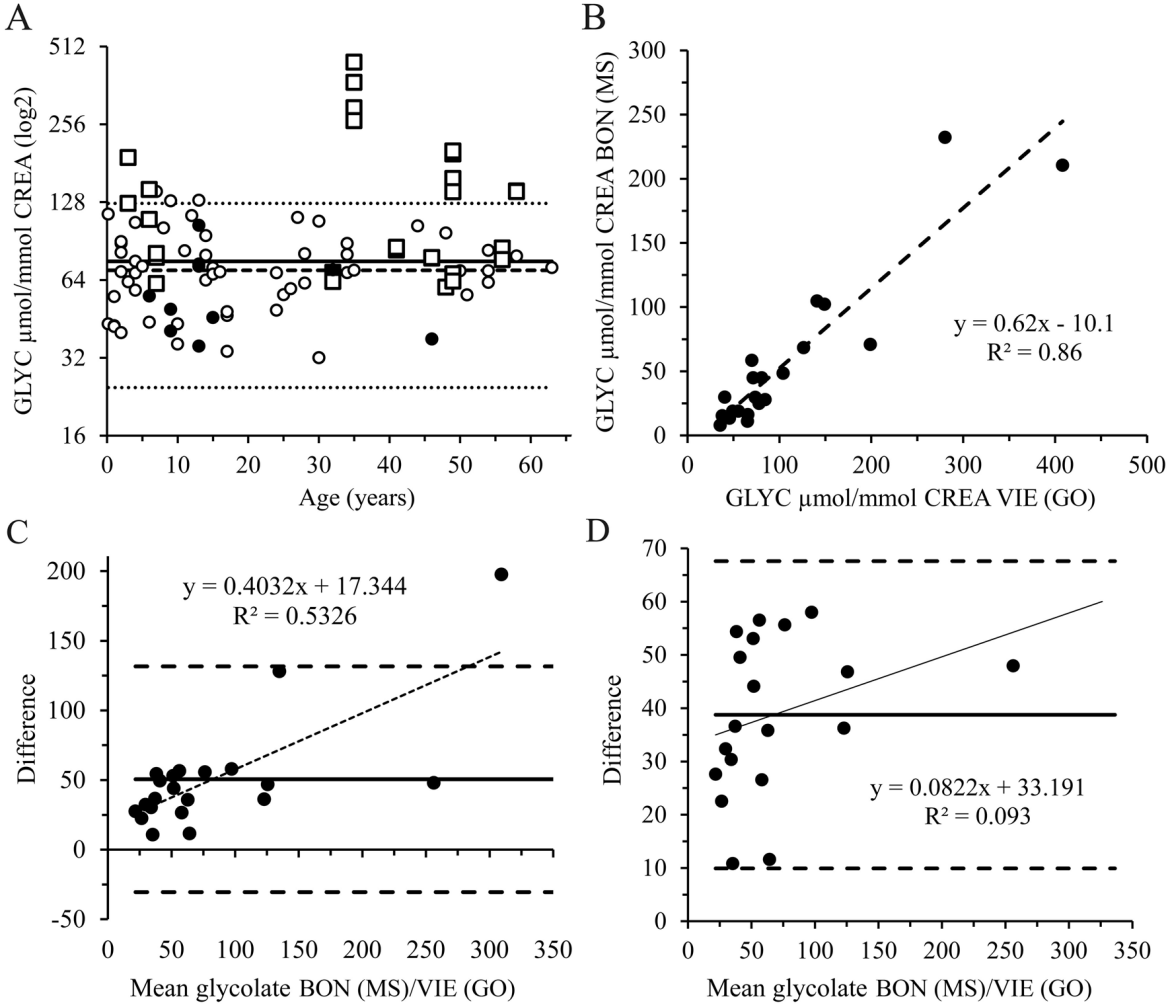
Determination of glycolate concentrations in healthy individuals and primary hyperoxaluria patients using the GO assay in comparison with IC-MS data. **A** Glycolate (GLYC) concentrations of 57 healthy volunteer (HV) urine samples (open circles) with the mean (solid line), median (dashed line) and the upper and lower 95% prediction intervals (dotted lines); The filled circles represent PH2 (*n* = 2), PH3 (*n* = 5) and 2 secondary hyperoxaluria samples with PH1 data (*n* = 12) shown as open squares; The PH1 samples have been measured twice within 2 weeks and the means of duplicate determinations of both measurements are shown partially almost completely overlapping; The mean/median CV of the 12 repeat samples is 7.4/6.6% respectively; **B** The glycolate concentrations of 21 urine samples of the different PH patients measured using MS in Bonn (BON) are plotted against the data measured using the GO assay in Vienna (VIE); The correlation coefficient R is 93% and *p* value of the regression 1.9E-09; **C** Bland-Altmann plot of all 21 differences (y-axis) versus the mean of GLYC concentrations measured in Bonn and Vienna including the mean (solid line; 50.6) and the 95% CI calculated using 1.96 * SD (SD = 41.33) of the differences (dashed lines); The two highest values were deemed outliers following the Grubbs test results and were removed to obtain a normal distribution using the Shapiro–Wilk normality test; **D** Bland-Altmann plot of the remaining 19 GLYC concentration differences including the mean (solid line) and the 95% CI (dashed lines); The correlation coefficient R^2^ is less than 10% and the regression line is not significant (*p* = 0.2); The *p* value of the regression increased more than 1100-fold; The mean (38.8) and the SD (14.32) of the differences are reduced 23/ 65% respectively after removing the two outliers; CREA = creatinine

We measured glycolate concentrations in 57 HVs and calculated the 95% prediction intervals (Fig. 4A). The glycolate concentrations of 12 PH1, 2 PH2 and 5 PH3 patients (Cohort_3_PH) are also shown in Fig. 4A. The PH1 urine samples were remeasured one week later and both duplicate measurements are shown. The mean/median CV comparing the two measurements was 7.4/6.6% respectively. Urine samples in Cohort_3_PH were spontaneously collected during routine outpatient visits and immediately frozen without addition of any preservatives. All 57 control spot urine samples (Cohort_0_HV) have been similarly collected. A short description of the different cohorts used in this study was added to the Supplement (Supplementary Tables S4 to S7). The correlation coefficient between the glycolate concentrations measured using the GO assay and IC-MS for Cohort_3_PH is 93% with a *p* value < 0.00001 (Fig. 4B). The IC-MS urine samples were collected at home, preserved with acid and sent to the analysis laboratory. The limits of agreement (LOA) in the Bland–Altman plot are acceptable meaning within the 95% CI after removing two outliers as determined using the Grubbs’ outlier test (Fig. 4C, D). After excluding the two outliers the data distribution did not deviate from a normal distribution using the conservative Shapiro–Wilk normality test.

We also measured glycolate concentrations in two additional PH cohorts, which were collected over 24 h at home. Cohort_1_PH was mainly preserved using 60 mM hydrochloric acid with some samples also containing a 1 in 100 dilution of Thymol 5%. For Cohort_2_PH only Thymol 5% was used for preservation. Thymol 5% starting between 0.05% and 0.1% shows significant GO inhibition interfering with measurements (Supplementary Fig. S5). We quantified the thymol concentrations in these urine samples and an increased correlation coefficient (from 73 to 93%) and an 11-fold decreased *p* value (from 0.0082 to 0.00074) comparing the glycolate concentrations determined using the GO assay with an IC-MS method can be obtained using multiple regression including measured thymol concentrations. These data are described in the Supplement (Supplementary Figs. S7–S9).

## Discussion

In 2019, about 140.1 million babies were born worldwide. Cochat and Rumsby [1] published that the life-birth incidence of PH1 in a European cohort is approximately 1 in 120,000 and therefore, it is likely that in 2019 worldwide 1168 newborns had PH1 mutations and will sooner or later present with clinically observable consequences of a PH1 mutation. Unselected newborn screening is unlikely an option to efficiently detect PH1 cases and is not recommended.

A technically simpler diagnostic method might nevertheless facilitate the rate of detection of PH1 cases throughout the world. Current state-of-the-art IC-, GC- and LC–MS/MS-based or similar methods are unlikely available in all areas of low income countries. Here, we report a simple, robust and inexpensive method to measure glycolate in urine samples of possible PH1 patients. This assay can be readily implemented in clinical laboratories worldwide, because only two simple and inexpensive chemicals (*ortho*-aminobenzaldehyde and glycolate as standard), recombinant GO, and a spectrophotometer or microplate reader with an absorption measurement module are necessary. We expressed recombinant mouse GO in *E. coli* followed by simple affinity purification using the attached hexa-histidine tag with high yield and low cost. Ready to use recombinant standardized GO might be deposited in a non-commercial setting for worldwide distribution. Unfortunately not all PH1 patients show elevated glycolate concentrations and therefore normal glycolate level does not exclude PH1. Other diagnostic tests including genetic and repeated urine analysis are necessary for a definite diagnosis. The GO assay might be also used in patients with possible ethylene glycol intoxication, because they show highly elevated plasma and urinary glycolate concentrations [30, 31]. Finally, the partially highly elevated plasma and urinary glycolate concentrations can be analyzed in HV and PH1 patients after administration of Lumasiran, an siRNA-based approved inhibitor of human GO [9, 12, 32].

There are three basic ways for GO assay interference. First, endogenous urinary aromatic and aliphatic aldehydes fuse with oABA and the added exogenous glycine generating CCMDQ or a similar dihydroquinazoline chromophoric structure. This option can be almost excluded because the concentration of aldehydes in urine is far below 1 µmol/mmol creatinine [33, 34]. Free glyoxylate is highly unstable and rapidly decays in urine even during storage at − 20 °C with preservatives (see below). Second, delta-1-pyrroline and delta-1-piperideine, the autocyclized products of putrescine and cadaverine deamination using diamine oxidase, delta-1-piperideine-6-carboxylate and delta-1-pyrroline-5-carboxylate, endogenous metabolites elevated in antiquitin deficiency and Type II hyperprolinemia respectively, fuse with oABA generating triple-aromatic ring dihydroquinazoline structures with absorption peaks between 430 and 460 nm [20, 35]. Plasma DAO concentrations except during pregnancy are less than 1 ng/mL generating very low amounts of delta-1-pyrroline and delta-1-piperideine excreted via glomerular filtration [36]. We measured a low background of less than a few µmol/mmol creatinine in HV urine samples using oABA [20]. Delta-1-pyrroline and delta-1-piperideine fuse with oABA and do not need glycine. Antiquitin deficiency and Type II hyperprolinemia are rare genetic diseases and the normal endogenous urinary concentrations of these compounds are below a few µmol/mmol creatinine and therefore at least 5- to tenfold below endogenous glycolate concentrations in healthy individuals [20, 35]. The high added exogenous glycine concentrations of 200 mM in the GO assay might also block fusion by rapidly interacting with oABA precluding fusion with these autocyclized protonated Schiff bases. We are not aware of other reactions of oABA with or without glycine generating chromophoric structures. This also explains the low mean/median (*n* = 34 urine samples) 440 nm peak and 90 min absorption units of 0.173/0.148 and 0.102/0.99 respectively in the absence of recombinant mouse GO.

Soda et al. [15] published that alanine, arginine, glutamine, phenylalanine and valine at 63 mM can react with oABA and glyoxylate but generate a 50 to 70% lower absorption signal compared to glycine. This interference can be also almost excluded because the sum of the mean concentrations of these amino acids in urine is less than 100 µmol/mmol creatinine and therefore at least 2000-fold below the added exogenous glycine concentration of 200 mM. In plasma the ratio of exogenous glycine to these amino acids is still approximately 200 indicating again low probability of significant interference (see below).

Third, compounds working as GO inhibitors or competing with glycolate for access to the GO active site will interfere in the assay. Although we tested only three compounds, oxalate, lactate and L-glycerate, Marangella et al. [19] tested 500 µM oxaloacetate, ketoglutarate, glutarate, L-lactate, pyruvate, oxalate, mesoxalate, L-citrate, L-glucose, L-ascorbate, L-tartrate, tartronate, malonate, maleate, malate, succinate and the main physiological L-amino acids in the GO assay for urine glycolate measurements and did not report relevant interference. They used GO to generate glyoxylate followed by fusion with phenylhydrazine and detection using reverse phase HPLC. Pyruvate is able to fuse with oABA and glycine generating a chromophore but the efficacy compared to glyoxylate is 30-fold less and therefore unlikely to interfere considering normal pyruvate concentrations of less than 10 µmol/mmol creatinine [15, 25]. Kasidas et al. [16] tested high concentrations of glyoxylic, malonic, uric, succinic, citric, formic, oxalic, acetic and hydroxybutyric acids in their GO assay for urine glycolate measurements and did not find relevant interference.

Although it has been described that human GO can oxidize lactate, the *K*_m_ ratio of glycolate to lactate is 295 and therefore glycolate is strongly preferred supporting the data of non-interference by high lactate concentrations [37]. In addition, the mean urinary lactate concentration in HVs is 12 µmol/mmol creatinine [25]. Glycolate oxidase can also use glyoxylate as substrate in the reverse reaction and this could interfere in the assay format [38]. This is rather unlikely, because the *K*_cat_/*K*_m_ ratio of human GO for glycolate to glyoxylate is 97 meaning that glycolate is the superior substrate, and oABA reacts immediately with glyoxylate blocking any further interaction with GO. Our non-interference data using L-glycerate, the metabolite abnormally elevated in the urine samples of PH2 patients, is supported by data using spinach GO. Huang et al. [39] showed that the *K*_m_ for L-glycerate is eightfold higher and the *K*_cat_ 20-fold lower compared to glycolate implying that glycolate is highly preferred over L-glycerate as substrate. It seems that recombinant mouse GO is highly specific for glycolate and cannot readily generate other aldehydes in urine consequently condensing with oABA and glycine generating a dihydroquinazoline chromophore.

The GO assay might also work measuring glycolate in plasma samples. Normal glycolate concentrations are approximately 7 µM but can increase 19-fold in PH1 patients or even 197-fold in PH1 patients undergoing dialysis [11, 19, 40]. Considering the extinction coefficient of CCMDQ of 2219 M^−1^ cm^−1^ every µM glycolate concentration increases the absorption by 0.0022 optical density (OD) units. A glycolate concentration of 100 µM would cause an absorption increase of 0.22 OD units, which is easily measurable after trichloroacetic acid (TCA) protein precipitation. Three plasma OD values at 440 nm were 1.11, 1.56 and 1.65 before and 0.129, 0.127 and 0.125 or approximately 9% after TCA precipitation (data not shown). A delta OD of 0.02 or 10 µM can be readily quantified [35]. We used TCA precipitation to measure low concentrations of diamine oxidase activity using oABA and delta-1-piperideine (oxidation product of cadaverine) or delta-1-piperideine-6-carboxylate condensation, which show an absorption peak at 460 nm [20, 35]. Therefore, two to fivefold elevated plasma glycolate concentrations should be readily measurable. Endogenous plasma concentrations of delta-1-pyrroline, delta-1-piperideine, delta-1-pyrroline-5-carboxylate or delta-1-piperideine-6-carboxylate are combined less than 10 µM, and therefore minimally interfere with the measurement of highly elevated plasma glycolate concentrations.

Free glyoxylate might be directly measured using oABA and glycine but plasma and urine must be immediately analyzed after collection. Petrarulo et al. [4] measured a half-life of 1.5 days of 200 µM glyoxylate spiked into urine during storage at − 20 °C without preservative or using 0.04% chlorhexidine and less than 1 day using 100 µM hydrochloric acid or pH < 2. Half-lives were less than 12 h at room temperature irrespective of using a preservative or not. We are not aware of plasma half-life studies with glyoxylate, but suspect similarly short half-life values.

Ortho-ABA-based glycolate quantification in plasma and urine might be improved using oABA derivatives, which still efficiently fuse with glycine and GO-generated glyoxylate but show higher extinction coefficients [41]. Glycine might be also exchanged with an amino group-carrying moiety possibly generating a triple aromatic ring structure or other complex structures with oABA and glyoxylate showing possibly not only an increased extinction coefficient but also fluorescence, which might significantly increase sensitivity.

## Conclusions

The GO assay converting glycolate into glyoxylate followed by fusion with oABA and high exogenous glycine concentrations can be easily implemented in most clinical laboratories worldwide and might help to identify PH1 patients as early as possible. Although low glycolate concentrations do not exclude PH1, relative increases or decreases during therapeutic trials or other interventions might be reflected in altered urinary glycolate concentrations, which can be readily measured with the GO assay based on a chemical condensation reaction described more than 85 years ago.

### Supplementary Information

Below is the link to the electronic supplementary material.

Supplementary file1 (PDF 1051 KB)

## Data Availability

The datasets used and/or analyzed during the current study are available from the corresponding author upon reasonable request.
