# Supplementary material for: Simple, fast and inexpensive quantification of glycolate in the urine of patients with primary hyperoxaluria type 1
Source: Urolithiasis. 2023 Mar 15;51(1):49. doi: 10.1007/s00240-023-01426-6 (PMC10017573; doi:10.1007/s00240-023-01426-6)
Supplement: Supplementary file 1 — Supplementary file1 (PDF 1051 KB) [file 240_2023_1426_MOESM1_ESM.pdf]

Supplement to

Simple, fast and inexpensive quantification of glycolate in the urine of patients with primary hyperoxaluria type 1

Thomas Boehm[1], Cristina Martin-Higueras[2], Eva Friesser[3], Clara Zitta[3], Silvia Wallner[3], Adam Walli[4], Katarina Kovacevic[1], Holger Hubmann[5], Kristaps Klavins[6], Peter Macheroux[3], Bernd Hoppe[2], Bernd Jilma[1]

[1]Department of Clinical Pharmacology, Medical University of Vienna, Vienna, Austria

[2]German Hyperoxaluria Center, Bonn, Germany

[3]Institute of Biochemistry, Graz University of Technology, Graz, Austria

[4]Laboratory Dr. Wisplinghoff, Forensic and Clinical Toxicology, Cologne, Germany

[5]Department of Pediatrics and Adolescent Medicine, Division of General Pediatrics, Medical University of Graz, Graz, Austria

[6]Rudolfs Cimdins Riga Biomaterials Innovations and Development Centre, Institute of General Chemical Engineering, Faculty of Materials Science and Applied Chemistry, Riga Technical University, Riga, Latvia

Correspondence

Thomas Boehm

Department of Clinical Pharmacology, Medical University of Vienna
Waehringer Guertel 18-20, 1090 Vienna, Austria
Tel.: +43-1-40400-49580, Fax: +43-1-40400-29980
Email: thomas.boehm@meduniwien.ac.at
ORCID Thomas Boehm https://orcid.org/0000-0002-8294-0797

More details about different “Materials and Methods” sections from the main manuscript

Generation of the dihydroquinazoline chromophore CCMDQ for mass spectrometry analysis

A Hamilton syringe pump was used to introduce samples into the mass spectrometer at a flow rate of 10 µL per minute. The mass spectrometer settings were as follows: positive ionization mode; source temperature: 150 °C; capillary voltages: 3.5 kV; desolvation temperature: 200 °C; cone voltage: 20 V. The mass spectra were acquired between 80 and 400 m/z with a scan speed of 1 second and an acquisition time of 30 seconds. Collision-induced dissociation using argon as the collision gas and collision energy of 30 arbitrary units was used to obtain MS2 spectra. Raw data files were used for preparing the figures in Microsoft Excel.

Expression and purification of recombinant mouse glycolate oxidase (GO)

A synthetic DNA fragment optimized for *E.coli* codon usage of mouse glycolate oxidase (mGO, Q9WU19, Hao1, EC 1.1.3.15) with flanked NdeI and XhoI restriction sites was cloned into pET24b with a C-terminal hexa-histidine tag. After transformation into *E. coli* Top10 cells individual clones were sequenced before transforming into *E. coli* BL21 Star™ (DE3) cells. Overnight cell cultures of *E.coli* were diluted to an absorbance of 0.1 at 600 nm and grown at 37 °C and 130 rpm for approximately 3 hours until an absorbance of 0.6 - 0.8 was reached. Heterologous protein production was induced by adding a final concentration of 0.1 mM isopropyl β-d-1-thiogalactopyranoside (IPTG) and protein expression was continued for 18 to 20 hours at 20 °C and 130 rpm. Cells were harvested by centrifugation at 16000 g for 10 minutes at 4 °C and were washed once with 0.7% saline solution.

Cell pellets were resuspended in lysis buffer (50 mM sodium dihydrogen phosphate (NaPi), 300 mM NaCl, 10 mM imidazole, pH 8.0) with 3 - 5 mL of lysis buffer per gram wet cell pellet. Before cell lysis a spatula tip (approximately 10 mg) of flavin mononucleotide (FMN) was added and cells were lysed using ultrasonication. The crude cell extract was cleared by centrifugation at 38000 g for 30 minutes. The supernatant was filtered through a 0.45 µm syringe filter and loaded onto a His-Trap Ni-NTA affinity column pre-equilibrated with 10 column volumes of lysis buffer. After washing with 5 column volumes lysis and 10 column volumes wash buffer (50 mM NaPi, 300 mM NaCl, 20 mM imidazole; pH 8.0) the GO protein was eluted with elution buffer (50 mM NaPi, 300 mM NaCl, 300 mM imidazole, pH 8.0). Target protein containing fractions were dialyzed overnight against storage buffer (100 mM NaPi, pH 8.3). Recombinant mouse GO was concentrated using Amicon

ultracentrifugation tubes with a molecular weight cut-off of 30 kDa and protein concentration was determined using the 448 nm extinction coefficient of GO of 9200 $M^{-1}cm^{-1}$[22]. Concentrated protein was flash-frozen as protein droplets in liquid nitrogen and stored at -80 °C until further use. In all presented GO assay data 1 µM (43 µg/mL) final GO concentration was used after thawing a protein droplet. At this concentration the GO absorption at 440 nm was 0.0120 and this value was subtracted from the samples containing GO. Thawed GO protein droplets were used only once within two hours after thawing. We also tested human and spinach GO in the same expression system, but mouse GO showed the highest expression levels and most stable protein expression and was selected for purification (data not shown).

Measurement of glycolate using mass spectrometry

The Dionex Integrion HPIC coupled with an ISQ EC single quadrupole mass spectrometer was equipped with a Dionex EGC III electrolytical KOH eluent generator cartridge with a Dionex CR-ATC 600 anion trap column, suppressor AERS 500e Integrion conductivity detector and a Dionex AS-AP autosampler. The Chromeleon chromatography data system software was used for analysis. Chromatographic separation was conducted at 30°C on Dionex IonPac AS11 (2×250 mm) and AG11 (2×50 mm) analytical and guard columns. All devices and software are from Thermo Fisher Scientific (Waltham, MA).

Urine collected at home over 24 hours was acidified with 6 mM HCl and transported to the laboratory within a few days. After arrival samples were analyzed within 24 hours in most cases. Urine samples (5 µl of a 1 to 100 dilution) were injected by the autosampler and a KOH multistep gradient was applied via the eluent generator cartridge with a flow rate of 0.3 mL/min. The gradient consisted of 5 to 40 µM (0–15 min), 40 to 100 µM (15–15.2 min), 100 µM (15.2–18 min), 100 to 5 µM (18–18.2 min) and 5 µM KOH (18.2–26 min).

Electrospray ionization in the negative mode was used with the following settings: vaporizer 500 °C, ion transfer tube 200 °C, source voltage 2500 V, source collision-induced dissociation (CID) 20 V, sheath gas 50 psig, auxiliary gas 5 psig and sweep gas off. Glycolate was detected using selected ion monitoring (SIM) at 75 m/z and quantified using a glycolate standard curve. Standards and controls were prepared using the certified reference material Supelco glycolate standard for IC (1000 mg/L glycolate in water, TraceCERT, 07391, Sigma-Aldrich). Urine samples were diluted 1 to 100 in 0.3 M boric acid (B6768 BioReagent, ≥99.5%, Sigma-Aldrich). The glycolic acid standard curve consisted of glycolic acid at 0, 0.2, 0.4, 1, 2 and 4 µM. Two quality control samples at 1.2 and 3 µM glycolate in 0.3 M boric acid were used. The method is specific and selective with the

calibration curve linear over the standard curve range tested using Mandel's F-test. Typical performance is reflected by a bias of -0.9 % with an intra- and inter-day precision of 9.8 % relative standard deviation (RSD) for both parameters. Samples of the ERNDIM External Quality Assurance Scheme for Special Assays in Urine are analyzed on a regular basis with satisfactory performance (https://www.erndim.org/).

Fragmentation MS2 analysis of the 235.071 m/z peak representing CCMDQ

Fragmentation of CCMDQ was performed using 20 V collision energy. The predicted and measured MS2 fragments are shown in Supplementary Fig. S1 and Table S1. The peak base in the stick diagram is 150 m/z (Fig. S1B). Other prominent peaks are at 106 (numbered 1 in Supplementary Fig. S1), 118 (2), 128, 145 (3), 168 (5), 191 (7) m/z. Five of these peaks including the peak base (106, 118, 145, 150 (4) and 191 m/z) have been predicted (Supplementary Table S1).

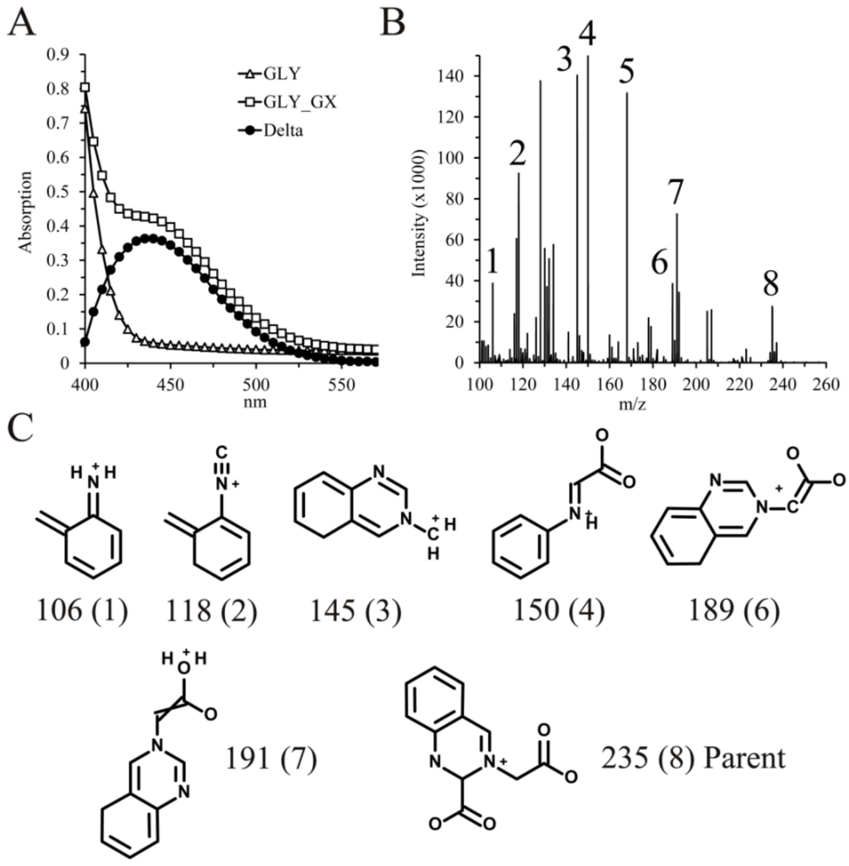

Fig. S1 Fragmentation of CCMDQ, the fusion product of glycine and glyoxylate with *ortho*-aminobenzaldehyde. **A** Absorption scan data from the samples used for MS analysis containing 100 mM glycine (GLY), 1 mM *ortho*-aminobenzaldehyde (oABA) with (GLY_GX; open squares) and without (GLY; open triangles) 300 µM glyoxylate (GX); The extinction coefficient was 2219 $M^{-1}cm^{-1}$; The delta of the two curves is shown as black circles; **B** Fragmentation stick diagram of the 235 m/z parent molecule (8); The chemical structures of all numbers except 5 are shown in (C); Several fragments have been predicted using the software at https://cfmid.wishartlab.com/predict; Measured and predicted fragments are shown in Supplementary Table S1; **C** Chemical structures of the predicted and detected fragments; Structures were drawn with PubChem sketcher V2.4.

Table S1 Predicted and measured MS2 fragments of the 235 m/z parent ion

Fragments	10 V*	20 V*	40 V*
Fr_x		104.0495	104.0495
Fr_1		**106.0651**	**106.0651**
Fr_2			**118.0651**
Fr_3	**145.0760**		**145.0760**
Fr_4		**150.0550**	
Fr_x		164.0706	
Fr_x			171.0553
Fr_6	**189.0659**	**189.0659**	**189.0659**
Fr_7	**191.0815**	**191.0815**	
Fr_y	217.0608	217.0608	
Parent (8)	235.0713	235.0713	235.0713

Measured fragments with peaks above 30000 intensity are shown in bold and with peaks above 60000 intensity in bold and underlined (Supplementary Fig. S1B); Only fragments >100 m/z are included; The measured base peak in the stick diagram was 150.0077 m/z (Supplementary Fig. S1B); Fragmentation prediction was performed using the software at https://cfmid.wishartlab.com/predict; The fragment numbering have been aligned with Supplementary Fig. S1; Fr_x/y represent signals only predicted but not measured or not a dominant peak and therefore not numbered. *For prediction 10, 20 and 40 V collision energy and for actual MS2 analysis 20 V collision energy were used.

The condensate of oABA, glyoxylate and glycine CCMDQ does not show any relevant fluorescence

Fusion of oABA with delta-1-piperideine (oxidation product using cadaverine as substrate and diamine oxidase) or delta-1-piperideine-6-carboxylate (a metabolite accumulating in patients with antiquitin deficiency) generates a fluorescent triple aromatic ring structure (Boehm 2020a and b). It was not known, whether the double aromatic ring fusion products of oABA with glycine and glyoxylate or other aldehydes are only chromophores or also fluorophores. We did not find any experimental evidence that the addition of glyoxylate to oABA and glycine generates relevant fluorescence (Supplementary Fig. S2). Addition of glyoxylate does not increase but decreases the relative fluorescence units (Supplementary Fig. S2). In contrast absorption increases 5- to 10-fold using 200 or 400 µM glyoxylate with glycine and oABA (Supplementary Fig. S3).

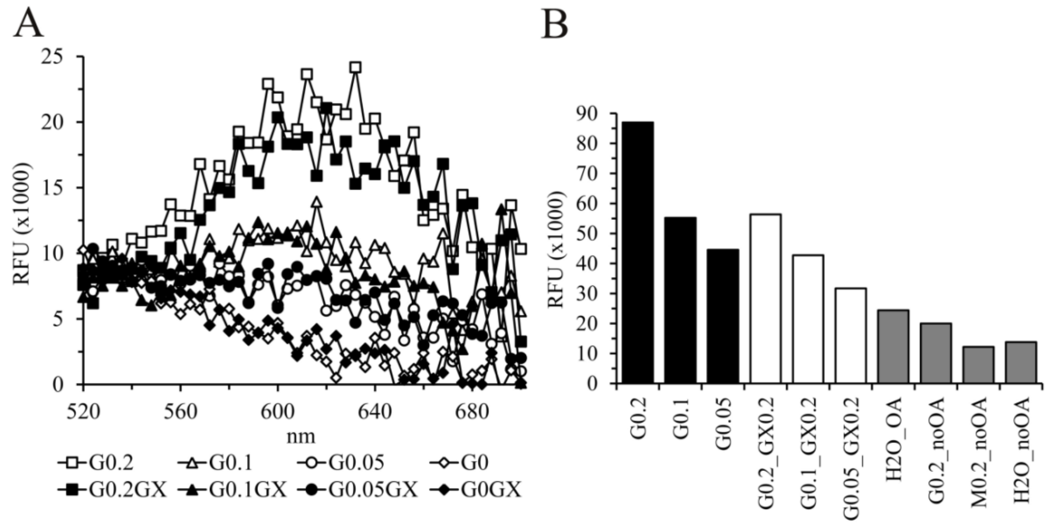

Fig. S2 CCMDQ does not show specific fluorescence.

A Emission scan after excitation at 440 nm of samples with 1 mM *ortho*-aminobenzaldehyde (oABA) and different glycine (G) concentrations (0, 0.05, 0.1 and 0.2 M) in the presence (filled symbols) or absence (open symbols) of 200 µM glyoxylate (GX); The relative fluorescence units (RFUs) have been normalized using buffer samples containing only 1 mM oABA; **B** Similar samples from an independent experiment were excited at 440/30 nm and the emission was measured at 620/40 nm using a custom-made filter cube; These wavelengths induce strong fluorescence in the triple aromatic ring structures generated from oABA and delta-1-piperideine or delta-1-pyrroline condensation; Addition of 200 µM glyoxylate (GX) reduced RFUs; G = glycine (200, 100 and 50 mM); OA = oABA; M = methylamine (200 mM).

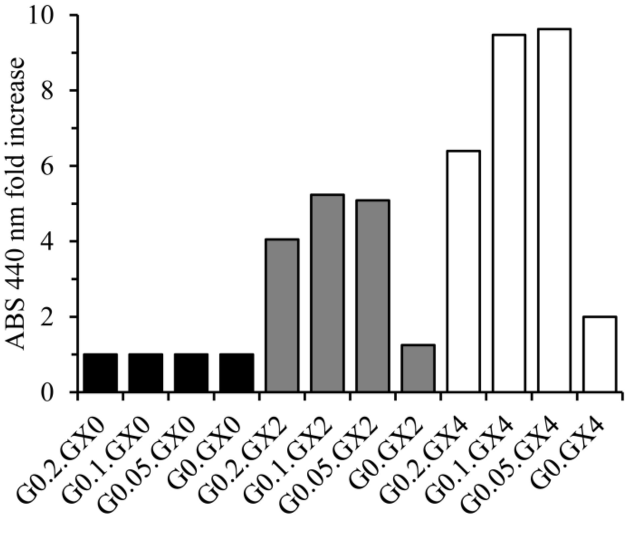

Fig. S3 Generation of CCMDQ induces a dose-dependent absorption increase.

Absorption data at 440 nm using 4 mM oABA, different glycine (G) concentrations (0, 0.05, 0.1 and 0.2 M) and 200 or 400 µM glyoxylate (GX) were normalized to samples without glyoxylate (GX0) and the fold-increases are shown; Black bars represent samples without GX normalized to 1; Grey bars indicated GX with 200 and unfilled bars with 400 µM.

Time course of the 440 nm absorption signals during condensation of glyoxylate with glycine and oABA in urine from healthy individuals

After adding oABA, glycine and glyoxylate to urine samples, peak absorption is reached within 10 minutes (Supplementary Fig. S4). After the peak the signal slowly degrades between 15 and 21 % in the four highest concentrations during the 60 minutes incubation time.

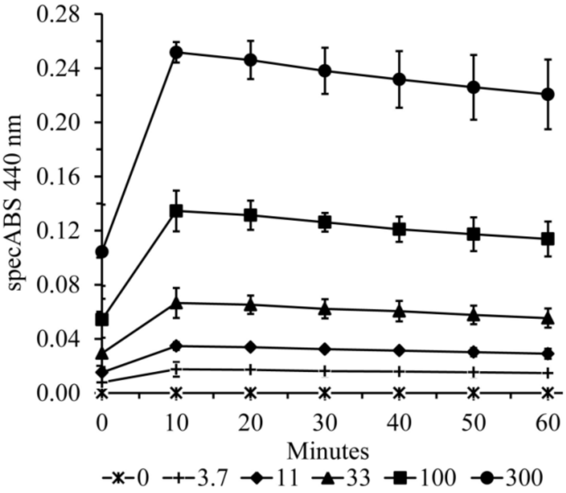

Fig. S4 Time course of specific absorption after spiking oABA, glycine and different glyoxylate concentrations into urine of healthy individuals.
Six glyoxylate concentrations (0, 3.7, 11, 33, 100 and 300 µM) were added to urine samples of three healthy volunteers (HV) together with 2 mM oABA and 200 mM ultrapure glycine and specific absorption (ABS) at 440 nm was normalized to no addition of glyoxylate (0); The means including the upper and lower 95 % confidence intervals of the different glyoxylate concentrations are shown; The peak means were used in Fig. 2A.

Minimal interference of high concentrations of oxalate, lactate and L-glycerate in the GO assay

Table S2 High concentrations of oxalate, lactate and L-glycerate do not relevantly interfere with glycolate quantification using the GO assay

	AUC	Percent	Absolute deviation	Deviation
OX250	20.6	107%	7%	-7%
OX125	20.5	107%	7%	-7%
OX63	20.4	106%	6%	-6%
LA250	18.2	94%	6%	6%
LA125	18.9	98%	2%	2%
LA63	18.4	96%	4%	4%
GLY250	20.1	105%	5%	-5%
GLY125	20.0	104%	4%	-4%
GLY63	18.7	97%	3%	3%
GLA250	19.3	100%	0%	0%
GO	5.0	**Mean**	**4.7%**	**-1.5%**
noGO	0.0	**SD**	**1.7%**	**5.1%**

The area under the curve (AUC) was calculated from the samples shown in Fig. 3B; OX = sodium oxalate, LA = sodium lactate, GLY = sodium L-glycerate; GO = mouse glycolate oxidase without exogenous substrate; noGO = only buffer control added to urine; SD = standard deviation; Numbers after the letters in the first column represent metabolite concentrations in µM.

High concentrations of thymol inhibit signal generation in the GO assay

The urine samples in Cohort_2_PH should have been collected using a 1 in 100 dilution of Thymol 5% (2-isopropyl-5-methylphenol in 100 % isopropanol; 12848.00100; Morphisto; Offenbach am Main, Germany). Nevertheless, patients were advised to dilute Thymol 5% during collection of the 24 hour urine samples at home. Therefore the final concentrations of thymol might vary considerably. Before we tested these urine samples in the GO assay we measured the influence of different thymol concentrations on the GO assay performance. If Thymol 5 % is properly diluted the final concentrations would be 0.05 % corresponding to 500 µg/mL or 3.3 mM thymol with 1% isopropanol. The GO inhibition data are summarized in Supplementary Fig. S5.

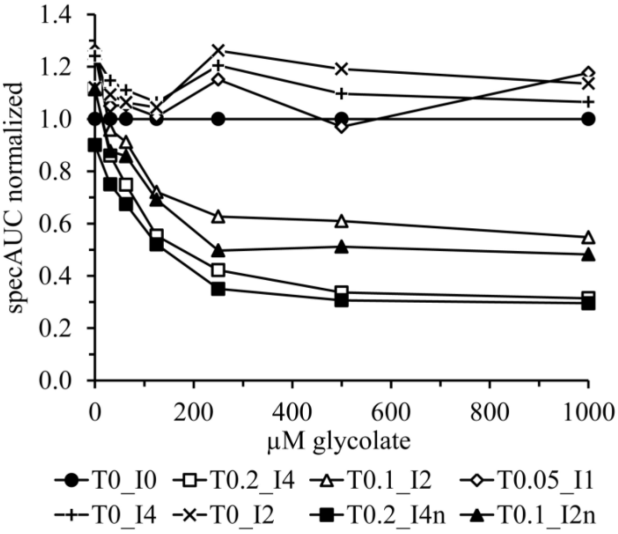

Fig. S5 Thymol inhibits recombinant mouse glycolate oxidase (GO).

Various glycolate concentrations were incubated with different thymol and isopropanol concentrations in the standard assay format using 1 µM GO, 200 mM ultrapure glycine and 4 mM oABA; T0 refers to no addition of thymol; T0.2_I4 refers to for example 0.2 % thymol and 4 % isopropanol final concentration; T0.2_I4n (filled squares) refers to T0.2_I4 (open squares) normalized to T0_I4; T0.1_I2n (filled triangles) referring to T0.1_I2 (open triangles) normalized to T0_I2; The AUCs of the specific absorption at 440 nm were normalized to T0_I0 (filled circles); T = thymol; I = isopropanol.

Recombinant mouse GO starts to be inhibited between 0.05 % and 0.1 % thymol, but inhibition is not linear, with higher glycolate concentrations more inhibited compared to lower concentrations. Different Thymol 5 % dilutions might have been used for some patients´ urine samples, and therefore thymol must be measured in each urine sample to somewhat estimate GO inhibition. We used the Gibbs method to quantify thymol including other phenolics in the relevant urine samples (Gibbs 1927). Gibbs reagent 2,6-dichloroquinone-4-chloroimide

condensates with phenol, phenolics (mainly p- and m-cresol) and also thymol generating a chromophore with absorption maximum at 610 nm.

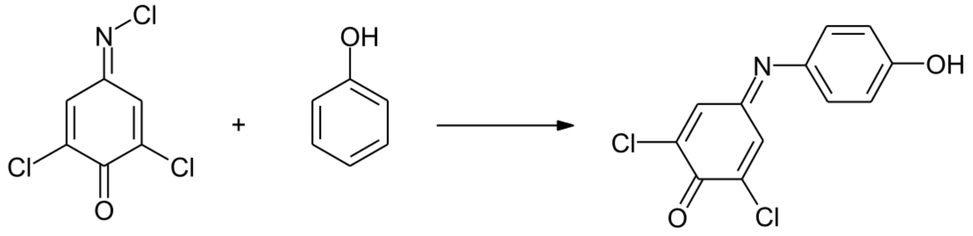

Fig. S6 Scheme of the Gibbs method

Phenol fuses with the Gibbs reagent 2,6-dichloroquinone-4-chloroimide generating a chromophore with absorption peak at 610 nm; The scheme is taken from https://commons.wikimedia.org/wiki/File:2,6-Dichloroquinone-4-chloroimide_reaction01.svg

Description of the Gibbs assay format used for thymol/phenolics quantification

142.5 µL of 50 mM borate buffer pH 9.4 was mixed with 142.5 µL 4 mM Gibbs reagent (2,6-dichloroquinone-4-chloroimide dissolved in ethanol; D6511; Sigma-Aldrich) and afterwards 15 µL urine samples or water were added. Urine samples were diluted 1 in 40 to 1 in 80 (final dilution factor) in the Gibbs assay. At this dilution the starting 60 mM HCl concentration is readily neutralized by the 50 mM borate buffer pH 9.0. The mixture was incubated at room temperature for 90 minutes with absorption measurements at 610 nm every 5 minutes. Thymol 5 % solution spiked into urine or water was used to derive the standard curve. An absorption scan between 400 and 700 nm showed a thymol concentration dependent increase in absorption with a peak at 615 nm (data not shown).

Urine did not relevantly influence the Gibbs method compared to water (Supplementary Fig. S7A) and a linear standard curve can be generated using 1 to 60 µg/mL thymol concentrations (Supplementary Fig. S7B and S7C). The signal is stable for at least 90 minutes. Based on the data shown in Supplementary Fig. S7D a wide range of Thymol 5 % concentrations have been added to the 24 hour urine collection samples. A thymol concentration of 0.05 % corresponds to 3.3 mM or 500 µg/mL. Some measured thymol concentrations were readily 10-fold higher in the 0.5 % (33 mM) range. At 0.2 % thymol recombinant GO was already significantly inhibited by 70 % at higher glycolate concentrations (Supplementary Fig. S5). The mean/median (SD) phenolics/thymol concentrations of 16 HV urine spot samples were 248/230 (141.4) µmol/mmol creatinine respectively (Supplementary Fig. S7D). A few thymol concentrations in PH samples were above 10 mmol/mmol creatinine (Supplementary Fig. S7D).

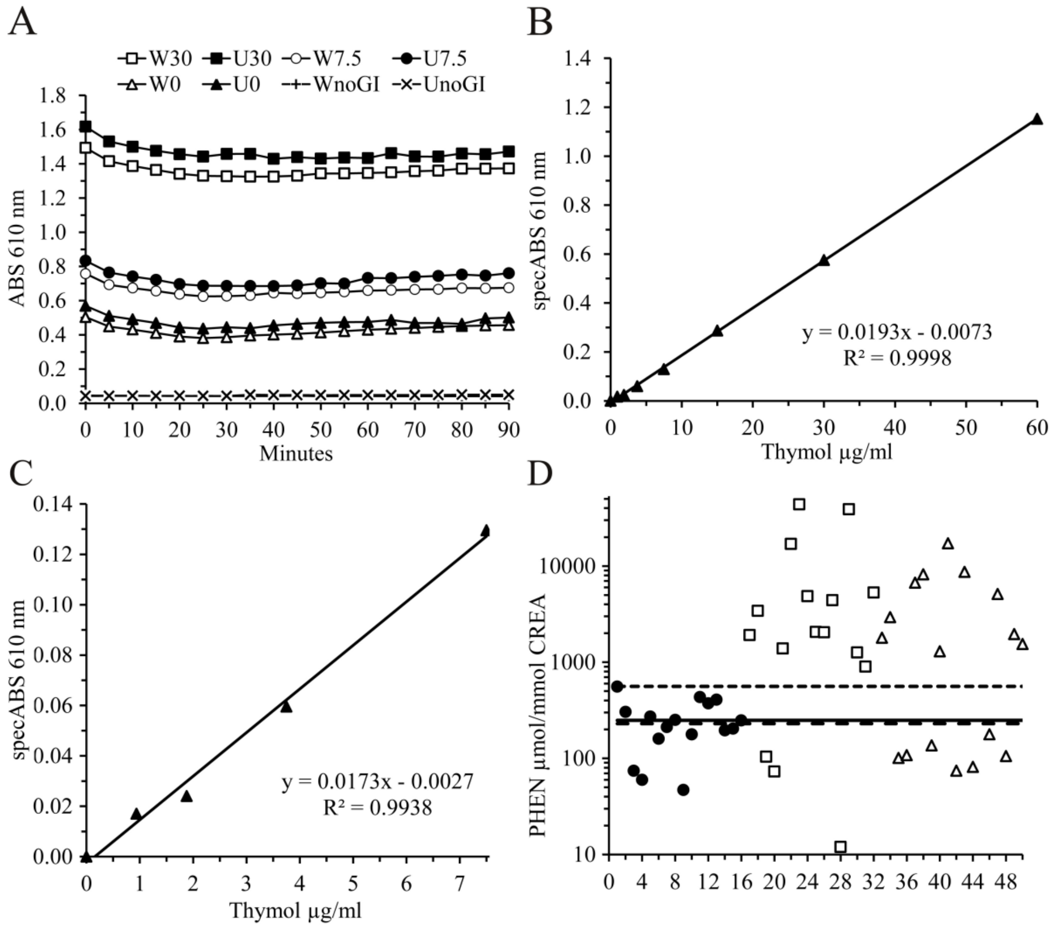

Fig. S7 The Gibbs assay can be used for thymol/phenolics quantification in urine samples.

A Time course of the absorption at 610 nm after spiking different thymol concentrations (0, 7.5 and 30 µg/mL) into urine (U; closed symbols) or water (W; open symbols); noGI means Gibbs reagent was not added; The mean of duplicate measurements are shown; **B** The curve shows the specific absorption (ABS) at 610 nm after spiking different thymol concentrations into urine of a HV; This is an example of a thymol standard curve using the Gibbs assay to quantify the phenolics (PHEN) concentrations in urine samples; **C** A detailed view of the standard curve shown in Panel B below 8 µg/mL thymol; **D** PHEN concentrations in µmol/mmol creatinine (CREA) in urine samples from 16 HV with no preservatives added before freezing (filled circles; 1 to 16; mean/median 248/230 with upper 95% prediction interval [dashed line] at 560), 16 collection urine samples (Cohort_2_PH open squares; 17 to 32) preserved with Thymol 5 % and 18 collection urine (Cohort_3_PH; open triangles; 33 to 50) preserved with 60 mM HCl (acidified) and Thymol 5 %; All measurements were performed in duplicate.

Measurement of glycolate concentrations in Cohort_1_PH and Cohort_2_PH using the glycolate oxidase assay and IC-MS

Both cohorts are briefly described in Supplementary Table S5 and Table S6. The IC-MS method (glycolate µmol/mmol creatinine BON) used for these cohorts is described below.

Acidified samples can be measured with the GO method with a high correlation coefficient of 93 % and low p-value of < 0.0001 (Supplementary Fig. S8A). Samples containing mainly Thymol 5 % as preservative show a lower correlation coefficient of 73 % but still a significant *p*-value of 0.0082 (Supplementary Fig. S8B).

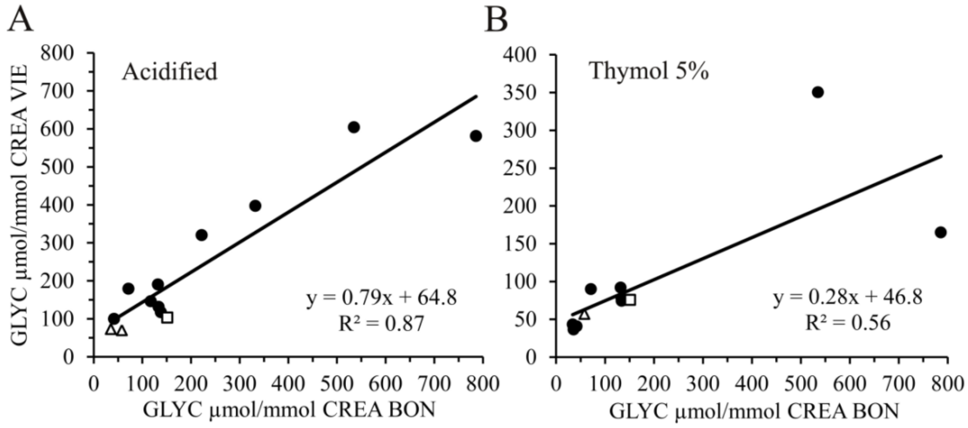

Fig. S8 Glycolate measurements in urine samples preserved with 60 mM hydrochloric acid and/or Thymol 5% **A** Regression analysis of glycolate (GLYC; µmol/mmol creatinine [CREA]) concentrations of 13 acidified samples (Cohort_1_PH; n = 18) measured in Vienna (VIE) versus Bonn (BON); For five samples no MS data from Bonn were available; The *p*-value is 3.02E-06 and multiple regression analysis including measured thymol/phenolics concentrations did not significantly influence the correlation coefficient or *p*-value (data not shown); **B** Regression analysis of glycolate (GLYC; µmol/mmol CREA) concentrations of 11 Thymol 5 % samples (Cohort_2_PH; *n* = 16) measured in Vienna (VIE) versus Bonn (BON); For five samples no MS data from Bonn were available; The *p*-value is 0.0082 and multiple regression analysis including measured thymol/phenolics concentrations reduced the *p*-value 11-fold to 0.00074 and increased the correlation coefficient of 73 % by 18 % to 93 %; The *p*-value for the thymol coefficient in the multiple regression was significant at 0.0025; In both Panels A and B the PH1 samples are shown as filled circles (●); PH2 as open triangles (Δ) and PH3 as open squares (□).

Below is a multiple regression analysis combining glycolate concentrations measured in Vienna and Bonn from Cohort_1_PH and Cohort_2_PH (Supplementary Fig. S9). Multiple regression including measured thymol/phenolics concentrations induced a 67-fold decrease in the *p*-value from 1.56E-05 to 2.35E-07 and a correlation coefficient increase from 76 % to 88 % (Supplementary Table S3).

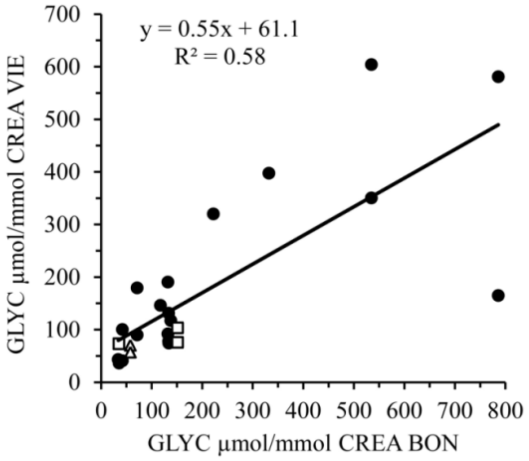

Fig. S9 Glycolate measurements combining Cohort_1_PH and Cohort_2_PH show high correlation after multiple regression using measured thymol/phenolics concentrations.

Regression analysis of the glycolate (GLYC; µmol/mmol CREA) concentrations of 24 samples combining Cohort_1_PH and Cohort_2_PH measured in Vienna (VIE) versus Bonn (BON); The *p*-value is 1.56E-05 and decreased 67-fold after multiple regression analysis including measured thymol concentrations to 2.35E-07 (Supplementary Table S3); The *p*-value for the Thymol 5 % coefficient was 4.36E-04; PH1 samples are shown as filled circles (●); PH2 as open triangles (Δ) and PH3 as open squares (□); CREA = creatinine.

Table S3 Influence of multiple regression analysis on *p*-value and correlation coefficients using the data shown in Supplementary Fig. S9.

	**w/o THYM**	**w/ THYM**	**Delta**
p-value	1.6E-05	2.4E-07	67-fold
R	76 %	88 %	+12 %
R^2	58 %	77 %	+19 %
R^2 adjusted	56 %	75 %	+19 %

w/o = without means regression analysis not including measured thymol/phenolics concentrations (THYM) using multiple regression; w/ = with means multiple regression analysis including measured thymol/phenolics concentrations.

Mass spectrometry method for glycolate measurements of Cohort_1_PH and Cohort_2_PH performed in Bonn

Urine samples were diluted 1 in 100 with 0.3 M boric acid and loaded into the autosampler. The glycolic acid standard curve was composed of glycolic acid at 2.5, 5, 10, 15 and 20 µM in 0.3 M boric acid. Two controls at 5 and 15 µM were also used. For measurements the Dionex ICS-2100 Ion Chromatography System (Thermo Fisher Scientific; Waltham, MA), an MSQ Plus^{TM} single quadrupole mass spectrometer (Thermo Fisher Scientific; Waltham, MA) and the Chromeleon^{TM} chromatography data system software was employed (Thermo Fisher Scientific; Waltham, MA). Glycolate was detected using selected ion monitoring (SIM) between 74.9 and 75.09 m/z and quantified using the glycolate standard curve.

Cohorts of urine samples used in this study

Table S4 Cohort_0_HV: n = 57; Spontaneous spot urine samples with no addition of preservatives; Immediately frozen in single use aliquots and stored at -80 °C.

Subject ID	Creatinine mM	Age	Glycolate µmol/mmol creatinine
GH24	1.8	0.2	115
GH26	1.3	0.2	43
GH03	0.7	1	55
GH04	1.9	1	43
GH13	0.7	1	42
GH02	1.5	2	89
GH05	3.1	2	40
GH06	1.3	2	90
GH07	7.7	2	82
GH21	1.9	2	69
H39	7.3	3	63
GH09	8.6	4	58
GH16	5.7	4	68
GH18	5.6	4	76
H38	4.9	4	107
GH14	3.3	5	72
GH20	0.9	6	44
GH10	4.5	7	141
GH11	7.6	8	102
GH01	4.8	9	129
GH22	11.1	10	36
GH23	3.0	10	43
GH27	4.8	11	83
GH17	6.7	12	114
GH25	5.7	13	72
H47	12.2	13	130
GH12	27.0	14	64
GH15	13.1	14	95
H46	23.7	14	80
GH19	4.2	15	71
H45	13.4	15	67
H44	19.9	16	69
GH08	12.6	17	47
H36	22.4	17	34
H42	2.6	17	48

KK55	2.7	24	68
H34	8.6	24	49
H35	1.5	25	56
H33	3.7	26	59
KK65	4.3	27	112
KK56	3.8	28	81
KK57	17.6	28	62
KK53	3.8	30	32
KK70	4.4	30	108
KK60	3.9	34	68
KK66	5.0	34	89
KK69	9.2	34	80
KK67	3.1	35	70
KK68	17.1	44	104
KK71	11.4	48	97
KK64	10.5	50	70
KK58	2.7	51	56
KK54	4.3	54	69
KK61	9.8	54	83
KK62	3.9	54	63
KK59	5.9	58	79
KK63	10.5	63	72

Table S5 Cohort_1_PH: *n* = 18; Acidified with 60 mM HCl; Partially also addition of 1 in 100 Thymol 5 %; 24 hour collection urine; Collection period 2019/2020.

Patient ID	Creatinine mM	Age	PH Type	Glycolate (µM) Bonn
1	3.4	9	PH2	195
2	4.5	16	PH1	318
3	3.2	12	PH3	485
4	9.1	16	PH1	1212
5	2.1	12	PH3	na
6	1.5	8	PH3	na
7	1.3	4	PH1	176
8	5.4	4	PH1	na
9	2.1	21	PH1	86
10	6.6	19	PH1	na
11	0.8	4	PH1	639
12	4.0	21	PH1	1342
13	2.7	19	PH1	605
14	2.0	16	PH1	238
15	2.0	40	PH1	na
16	4.8	38	PH2	169
17	11.1	13	PH1	1462
18	3.4	19	PH1	1811

PH = primary hyperoxaluria; na = not available

Table S6 Cohort_2_PH: *n* = 16; Addition of 1 in 100 Thymol 5 %; 24 hour collection urine; Collection period 2019/2020.

Patient ID	Creatinine mM	Age	PH Type	Glycolate (µM) Bonn
HB01	3.4	9	PH2	195
HB02	4.5	16	PH1	318
HB03	3.2	12	PH3	485
HB04	9.1	16	PH1	1212
HB05	5.4	4	PH2	na
HB06	2.1	21	PH1	86
HB07	0.7	4	PH1	639
HB08	2.0	40	PH1	na
HB09	11.1	13	PH1	1462
HB10	3.4	19	PH1	1811
HB11	2.1	21	PH3	na
HB12	2.1	9	PH3	na
HB13	0.8	1	PH2	na
HB14	5.0	64	PH1	180
HB15	7.4	29	PH1	997
HB16	4.8	20	PH1	162

PH = primary hyperoxaluria; na = not available

Table S7 Cohort_3_PH: *n* = 25; spontaneous spot urine samples with no preservatives added; Immediately frozen and combined shipment to Vienna; Collection period within 4 months in 2021. IC-MS/MS glycolate measurements in the Wisplinghoff laboratory was performed on acidified 24 hour collection urine as described under Material and Methods in the main manuscript.

Patient ID	Creatinine mM	Age	PH Type	Glycolate (µM) Bonn
W01	8.9	13	PH3	167
W02	7.6	15	PH3	143
W03	9.0	13	PH3	269
W04	14.5	13	PH3	115
W05	7.3	32	PH3	99
W06	5.2	10	PH2	153
W07	4.4	10	PH2	214
W08	9.1	9	secH	535
W09	11.7	9	secH	179
W10	3.3	35	PH1	685
W11	1.2	7	PH1	53
W12	1.4	7	PH1	na
W13	5.3	49	PH1	58
W14	0.8	3	PH1	na
W15	6.1	49	PH1	433
W16	3.5	41	PH1	99
W17	4.2	35	PH1	973
W18	0.4	3	PH1	na
W19	2.0	56	PH1	89
W20	3.2	49	PH1	330
W21	1.6	58	PH1	168
W22	3.1	32	PH1	50
W23	3.1	48	PH1	na
W24	6.1	46	PH1	153
W25	2.0	6	PH1	134

PH = primary hyperoxaluria; na = not available; secH = secondary hyperoxaluria.

Supplement references

Boehm T, Karer M, Gludovacz E et al (2020a) Simple, sensitive and specific quantification of diamine oxidase activity in complex matrices using newly discovered fluorophores derived from natural substrates. Inflamm Res 69(9):937-950. https://doi.org/10.1007/s00011-020-01359-5.

Boehm T, Hubmann H, Petroczi K et al (2020b) Condensation of delta-1-piperideine-6-carboxylate with ortho-aminobenzaldehyde allows its simple, fast, and inexpensive quantification in the urine of patients with antiquitin deficiency. J Inherit Metab Dis 43(4):891-900. https://doi.org/10.1002/jimd.12214.

Gibbs HG (1927) Phenol tests: III. The indophenol test. J Biol Chem 72(2): 649-664. https://doi.org/10.1016/S0021-9258(18)84338-1.
